# A networked voting rule for democratic representation

**DOI:** 10.1098/rsos.172265

**Published:** 2018-03-07

**Authors:** Alexis R. Hernández, Carlos Gracia-Lázaro, Edgardo Brigatti, Yamir Moreno

**Affiliations:** 1 Instituto de Física, Universidade Federal do Rio de Janeiro, Rio de Janeiro, Brazil; 2 Institute for Biocomputation and Physics of Complex Systems (BIFI), Universidad de Zaragoza, Zaragoza, Spain; 3 Department of Theoretical Physics, Faculty of Sciences, Universidad de Zaragoza, Zaragoza, Spain; 4 ISI Foundation, 1, Turin, Italy

**Keywords:** physics of social systems, social networks, mathematical modelling, e-Democracy

## Abstract

We introduce a general framework for exploring the problem of selecting a committee of representatives with the aim of studying a networked voting rule based on a decentralized large-scale platform, which can assure a strong accountability of the elected. The results of our simulations suggest that this algorithm-based approach is able to obtain a high representativeness for relatively small committees, performing even better than a classical voting rule based on a closed list of candidates. We show that a general relation between committee size and representatives exists in the form of an inverse square root law and that the normalized committee size approximately scales with the inverse of the community size, allowing the scalability to very large populations. These findings are not strongly influenced by the different networks used to describe the individuals’ interactions, except for the presence of few individuals with very high connectivity which can have a marginal negative effect in the committee selection process.

## 1. Introduction

The selection of an exemplar group of representatives to make decisions on behalf of a larger community is a widespread and critical problem for human societies [1,2]. Examples can be found in the election of a legislative assembly in indirect democracies, in the elections for the trade union, for supervisory or faculty board, for executive officers or non-governmental organization boards. The most widely used electoral systems can be classified into one of the following groups: first-past-the-post, two-round systems, proportional representation, ranked voting or in a mix of two or more of the previous groups [3–8]. In general, these systems seek to strike a balance between representativeness and effectiveness [9]. In the vast majority of electoral systems, including fully proportional representation systems [10,11], representatives gain a power of representation that is not completely proportional to the number of voters they represent, but rather the result of a given granularity. Interestingly, this problem is also relevant for artificial systems, such as software multiagent systems, i.e. in recommendation systems [12,13], mobile networks [14] and election-based mechanisms [15] for distributing data over an overlay P2P network [16].

In the last years, a general interest in the quantitative description, based on mathematical models, of social processes related to election procedures increased significantly. One of the reasons can be related to the successful introduction of agent-based modelling. In fact, agent-based models (ABM) provide a flexible and powerful theoretical framework for describing these phenomena [17–19]. One of the first opinions ABM was proposed by Clifford & Sudbury [20]. Although, at first, it was designed as a theoretical tool for studying the competition of species, it has been named voter model for the natural application to the dynamics of voting systems [21]. The voter model has been studied under different topologies [22–25] and conditions [26–28]. Nevertheless, although it can capture statistical features of real-world elections [29], its intrinsic limitations, such as the consideration of a unique cultural variable that can only take two values, restrict its applicability. Subsequently, new social models have been introduced to study the voting dynamics [30–32].

In this work, we analyse a quite different problem related to election procedures, which has not been addressed in previous works which use ABM. In fact, we study the general problem of selecting a group of candidates that best represents the voters. We consider systems where each voter is allowed to vote for only one candidate and the elected are the ones who obtain a better rank among their counterparts. In particular, we focus on the case of multi-winner elections, that is choosing a collective body of a given size (a committee). We model an idealized situation where voters are rational individuals, which means that they make a decision to maximize their representation, and they present a general knowledge of the candidates and direct access to them.

In classical elections, a fixed number of candidates participate and voters rank the candidates expressing their preferences. We introduce a new formal model, where the list of candidates is not fixed in advance, but they emerge as a self-organized process controlled by the voting rules. Moreover, voters express not preferences, but opinions, which determine their indications about whom they would like to see as their representative.

Our model introduces new mechanisms which give a fundamental importance to the accountability of the elected committee. The connection between representatives and constituents is fundamental and it is the basis for accountability, allowing us to check for incompetence and corruption. In classical voting systems, this link can be generally insured only for a small-sized community. In particular, for the national legislative assembly, it can be partially controlled by the small size of the electoral district. Our model introduces a radical difference for obtaining an efficacious accountability. In fact, it takes into account individuals’ first-hand trust relationship as a key ingredient to determine the elected representatives. Votes are assigned on the basis of a self-declared confidence circle, which is a network of trusted individuals which can be implemented on an online platform.

After having implemented this new voting rule, its effects are tested modelling the behaviour of the selected committee. The committee runs a series of ballots making choices about different issues. The quality of the elected committee is numerically assessed based on how much their final decisions are consistent with the personal opinions of the community. Note that many works study scenarios where representatives make decisions which are compared with some objective truth [1,33]. By contrast, as in [34], our approach is interested in discriminating the selected boards which best represent the community opinions.

## 2. The model

The system is composed of a population of *N*_e_ electors and an internet-based platform. The platform allows the voters to self-declare who belongs to their confidence circle, which is a network of trusted individuals. The same platform is used by voters to manifest their opinions on *N*_*i*_ issues. Issues are organized in questions which can be defined by a committee or by means of a self-organized process internal to the community. The answers of each individual *j* are organized in a vector *v*^*j*^. The vector is composed of *N*_*i*_ cells and each cell can assume the value 1 if the answer is positive, −1 if it is negative or 0 if the question is left unanswered.

The following step allows us to find the better representative for each confidence circle. First, we consider an individual *j* and we compute the vectors overlaps with all his neighbours *k*. This is obtained using the following expression:

2.1 $$v^{\;j}*v^{k}=\frac{\sum_{m=1}^{N_{i}}(v_{m}^{\;j}\cdot v_{m}^{k})\delta (v_{m}^{\;j},v_{m}^{k})}{\sum_{m=1}^{N_{i}}{\left(v_{m}^{\;j}\cdot v_{m}^{k}\right)}^{2}},$$

where the numerator counts the number of questions answered in the same way (only yes or not) and the denominator counts the number of questions answered simultaneously by both individuals; *δ* stands for the Kronecker delta which is 1 if $v_{m}^{j}=v_{m}^{k}$ and 0 otherwise. Each individual *j* will indicate as his representative the individual *k*′ for which *v*^j^*v^*k*′^ is maximum. In the case where more than one individual generates the same maximum overlap value, the individual with a higher connectivity is chosen as the representative. For the exceptional case when also the connectivity is equal, the representative is randomly selected between the similar ones.

**Figure 1.**
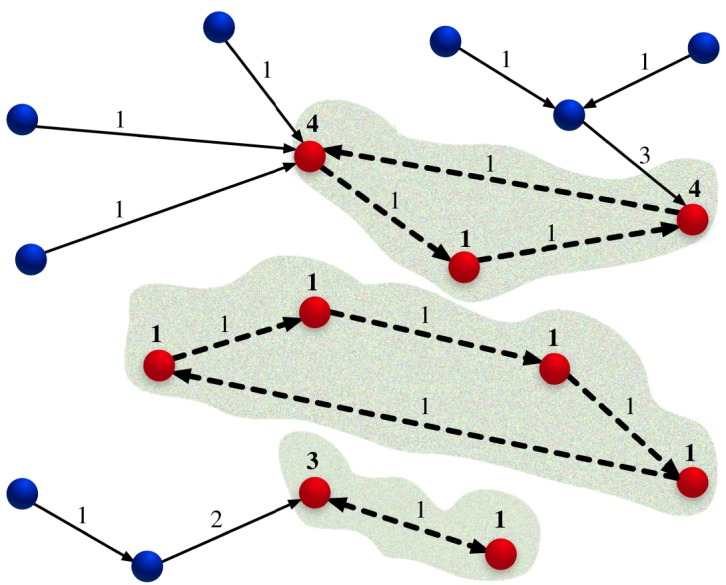
Schematic of the vote process. Nodes stand for the individuals; the red ones belong to a cycle and will be confirmed as representatives if they collect more votes than the established threshold. The big numbers associated with the nodes represent the received cumulated votes. Arrows stand for the indication of each individual and the small numbers associated with them represent the number of transferred votes. Dotted arrows belong to a cycle, where there is no cumulative transfer of votes.

After the selection of the representative *k*′ for every voter *j*, as constrained by his confidence network, the final step consists in choosing the aggregate of representatives of the entire community. To this end, we construct a directed graph where a node represents each individual and a directed link connects the individual with his personal representative. In this graph, which, in general, can be composed by different disconnected clusters, cycles are present. They represent individuals that have been mutually indicated by themselves. As all the individuals outside the cycles are represented by the individuals belonging to them, individuals who belong to cycles are the proper potential representatives for the community (figure 1).

As a final step, among the individuals belonging to a cycle, only the ones with a number of votes larger than a threshold *θ* are indicated as representatives. Votes are counted considering the cumulative flow defined by the directed graph. If the individual *j* is pointing to *z*, *z* receives all the votes previously received by *j* plus one. This flow of votes is computed only following links outside the cycles. Inside the cycles, only the single vote of an individual is counted. In this way, the number of representatives is reduced and results to be a fraction of the total individuals which belong to a cycle.

## 3. Results

### 3.1. General analysis

The individuals’ opinions in relation to the selected issues are randomly generated with the following rule: given an issue *i*, an individual does not present an opinion (*v*_*i*_=0) with probability $\frac{1}{3}$. The probability to have an opinion *v*_*i*_=+1(−1), is $\frac{1}{3}+\epsilon_{i}$ $\left(\frac{1}{3}-\epsilon_{i}\right)$, where *ϵ*_*i*_ is a random variable following a normal distribution with mean value equal to zero and *σ*^2^=0.05. Such a simplified characterization of the single opinion presents some contact with real political opinions which, frequently, are polarized, presenting a natural bimodality of preferences in political and economical issues [35].

The confidence circle of each individual is modelled generating a network where nodes represent individuals and links the trust relationships present in the community. The confidence circle of an individual is obtained selecting a node and considering its first neighbours. Note that an important simplification of this approach is the fact that it generates individuals with symmetric trust relationships. In the following analysis, three types of networks are considered. Homogeneous random networks, implementing the Erdös–Rényi model [36], where the degree distribution is peaked around a typical value 〈*k*〉, heterogeneous networks, using the Barabasi–Albert model [37], with a power-law degree distribution *P*(*k*)∝*k*^−3^, and networks with the small-world property using the Watts and Strogatz model [38]. Our aim is not to model specific aspects of a real social network, but to use simple examples just to discuss the possible influence of some relevant network properties (such as the heterogeneity in the degree distribution, the average degree and the small-world property), on the behaviour of our model.

The exploration of the system behaviour can be obtained considering two fundamental observables. The first one is the normalized committee size which is measured as the ratio between the number of elected individuals (*E*) and the total number of individuals of the community: *F*_rep_=*E*/*N*_e_. To have manageable committees, a small value of *F*_rep_=*E*/*N*_e_ is preferable. The second one is the representativeness. This is defined measuring the fraction of decisions expressed by the elected committee (*e*_*j*_) which matches with the community decisions (*c*_*j*_) over all the considered *N*_*i*_ issues: $R=\sum_{j=1}^{N_{i}}\delta (e_{j}-c_{j})/N_{i}$. Then, for *R*=1 we have a perfect committee, which make all the decisions in line with the popular will and for $R=\frac{1}{2}$ (for binary decisions) we have a non-representative committee, whose decisions are completely uncorrelated to the popular will.

The decision expressed by the elected committee is obtained through a majority vote where each representative’s vote is weighted by the numbers of popular votes he received in the election procedure. The community decision is obtained by a direct process (plebiscite), where every individual votes in accordance with the opinion expressed in his vector *v*^*j*^. Note that if the individual has no opinion on a particular issue, he abstains from voting.

**Figure 2.**
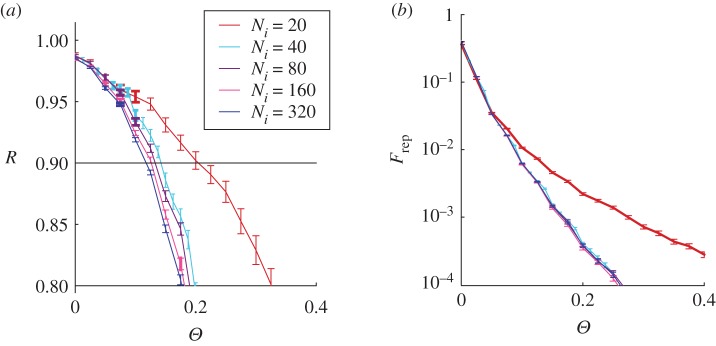
Representativeness (*a*) and the normalized committee size (*b*, semi-logarithmic plot) as a function of the threshold *Θ* for different values of the number of issues *N*_*i*_. Both panels correspond to *N*_e_=10 000 and confidence circles are defined from an Erdös–Rényi network with 〈*k*〉=40. Results are averaged over 100 different realizations.

In figure 2, we show the representativeness *R* and the normalized committee size *F*_rep_ as a function of the threshold *Θ*=*θ*/*N*_e_ for different values of the number of issues *N*_*i*_, as obtained in a typical system with confidence circles defined from an Erdös–Rényi network. As expected, the representativeness and the normalized committee size decrease when increasing the threshold value. In particular, the decrease of the normalized committee size is very fast.

For fixed threshold values, decreasing the number of issues, quite intuitively, increases the representativeness. Similarly, it increases the normalized committee size. This last effect is not obvious and it is produced by the fact that for a small number of issues the distribution of votes has a less pronounced peak and the threshold is not very efficient in selecting between the indicated individuals. Anyway, this tendency rapidly saturates and, for *N*_*i*_>40, the curves do not show any relevant dependence on this parameter.

**Figure 3.**
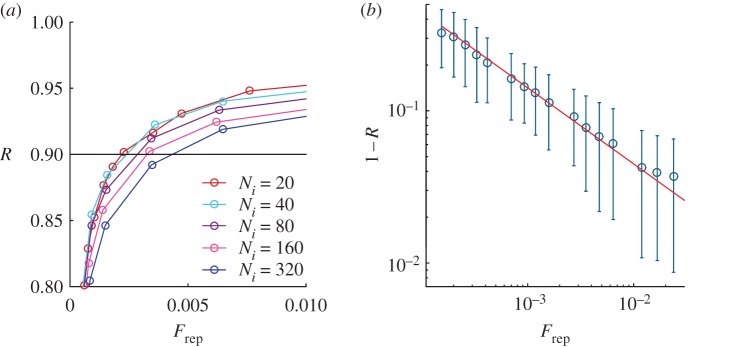
(*a*) Representativity versus normalized committee size, (*b*) logarithmic plot of 1−*R* versus normalized committee size for *N*_*i*_=40. The continuous line has slope $-\frac{1}{2}$. For both figures, *N*_e_=10 000 and confidence circles are defined from an Erdös–Rényi network with 〈*k*〉=40. Results are averaged over 100 different realizations.

The ideal committee corresponds to a small group of representatives which expresses a high level of representativeness. This is obtained by selecting an intermediate value for *Θ*, which can be identified seeking for a representativeness close to 0.9, and looking at the corresponding committee size. For this reason, in the following we will plot the representativeness versus the normalized committee size, which allows a clear visualization of this fundamental relationship. In figure 3, we can observe that for fixed values of *R*, the normalized committee size increases when the number of issues increases.

The relation between *R* and *F*_rep_, as reported in figure 3, can be characterized by a simple relation: $1-R\propto 1/\sqrt{F_{\mathrm{rep}}}$ of the obtained representativeness in relation to the ideal one scales as the square of the inverse of the normalized committee size. This means that, for example, for improving *R*−1 by a factor of 2, the number of elected must quadruple. This relation can be justified considering that 1−*R* is proportional to the error in the estimation of the mean opinion *v*_*i*_ (the result of the plebiscite) using a sample of size *F*_rep_. In the case where the estimation of the mean opinion uses an independent and identically distributed sample of size *n*, it is well known that the standard error of the sample mean scales as *n*^−1/2^. Interestingly, the same scaling law is preserved using our voting rule, which effectively can be seen as a particular data sample strategy.

**Figure 4.**
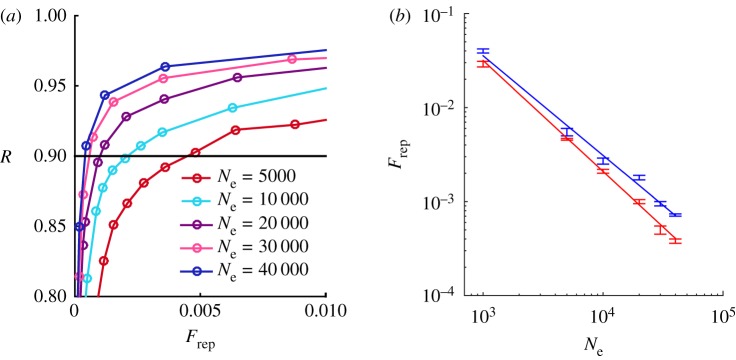
(*a*) Representativity versus normalized committee size for an Erdös–Rényi network. (b) Logarithmic plot of the normalized committee size fixing *R*=0.9, as a function of the number of electors. Data are well approximated by a power-law fitting (continuous lines). For the Barabasi–Albert network (blue) the power-law exponent is −1.06±0.05, for the Erdös–Rényi network (red) −1.18±0.03. The parameters used in the simulations are 〈*k*〉=40 and *N*_*i*_=40 . Results are averaged over 100 different realizations.

A similar analysis was conduced looking at the dependence on the system size *N*_e_ (figure 4). Surprisingly, fixing *R*, the committee size decreases with the system size. For example, for the parameters used in figure 4, a representativity of 0.9 is obtained with a committee of 29 members for a community of 1000 individuals, and with just 15 representatives for *N*_e_=30 000. In particular, fixing *R*=0.9, *F*_rep_ decreases approximately with the inverse of *N*_e_ using a Barabasi–Albert network, and even faster for an Erdös–Rényi network.

**Figure 5.**
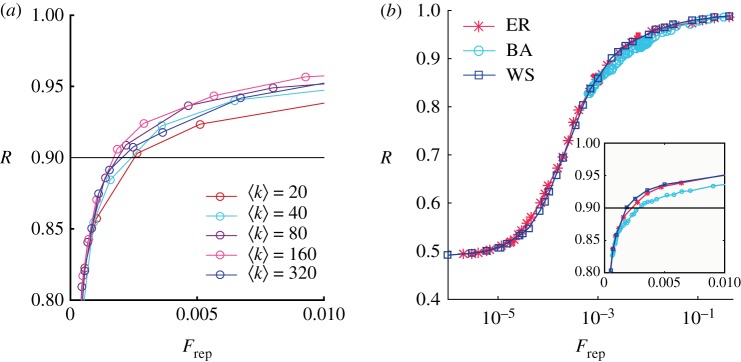
Representativity versus the fraction of elected citizens. (*a*) We consider an Erdös–Rényi network with different connectivities, *N*_e_=10 000 and *N*_*i*_=40. (*b*) We display the semi-logarithmic plot of three different networks: Erdös–Rényi (ER), Barabasi–Albert (BA) and Watts–Strogatz (WS), with *N*_e_=10 000, *N*_*i*_=40, 〈*k*〉=40. The Watts–Strogatz network has *β*=0.1. Results are averaged over 100 different realizations.

In figure 5, we can see that the representativeness is not strongly dependent on the connectivity of the network, and for 〈*k*〉>40 the curves present very similar behaviours. The heterogeneity in the degree distribution of the network seems to have a relative small impact on the results too, as it can be appreciated by comparing the results of the Erdös–Rényi network with the Barabasi–Albert one. Finally, the small world property of the Watts–Strogatz model does not influence our results.

**Figure 6.**
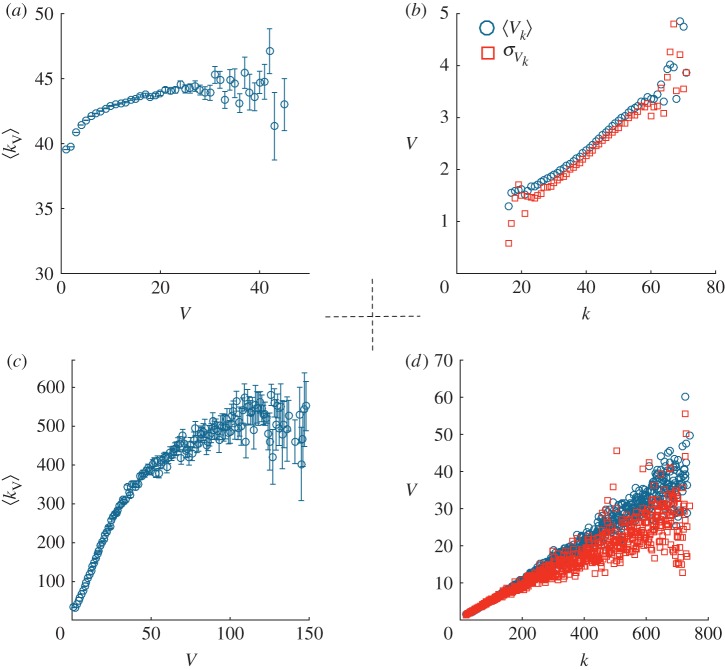
(*a*,*c*) Mean connectivity 〈*k*_*V*_〉 of an individual which received a number of votes *V* . (*b*,*d*) Mean number of votes 〈*V*_*k*_〉 collected by individuals with a given connectivity *k*. Data are obtained using 1000 different realizations in an Erdös–Rényi network (*a*,*b*) and a Barabasi–Albert one (*c*,*d*), with 〈*k*〉=40, *N*_*i*_=40 and *N*_e_=10 000.

These results are consistent with the inspection of the relation between the individual’s connectivity and the number of votes they received. As can be appreciated in figure 6, even if it is necessary a reasonable connectivity (higher than the mean value) to obtain votes and a higher connectivity increases the probability to obtain more votes; for Erdös–Rényi networks this effect is weak and not impactful. For the case of the Barabasi–Albert network, this effect is more relevant and probably it is responsible for the influence that this topology has on the behaviour of the representativity and normalized committee size (figure 5), determining a slightly weaker performance. In fact, it moderately decreases the representativeness for a fixed committee size, as higher connectivity generates a bias in the selection of the more representative individuals.

### 3.2. Comparison with other voting rules

In the following, we explore if our model, at least theoretically, selects committees who present a representativeness comparable with other traditional methods of body selection. Obviously, representativeness is compared among committees of the same size.

**Figure 7.**
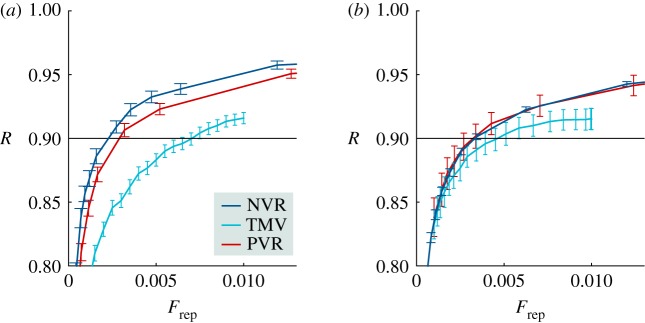
Representativity versus the fraction of representatives for the proposed networked voting rule (NVR), the traditional majority voting (TMV) and a perfect voting rule (PVR). Parameters: *N*_e_=10 000, 〈*k*〉=40, *N*_*i*_=40 (*a*) and *N*_*i*_=160 (*b*). For the TMV, we consider *N*_c_=100. See the main text for detailed explanations of the voting rules.

A first possible comparison is with a model of a traditional majority voting for the selection of representatives in a closed list of previously selected candidates. This is probably the most common practice in selecting committees. An example can be found in the election of legislative assembly with the system of multi-member districts. In our simulations, a list of *N*_c_ candidates is randomly selected among the community and each individual votes for the candidate who presents the higher overlap with its opinion vector. Decisions are taken with the same weighted voting rule. This modelling approach mimics a voter who presents a perfect knowledge of the candidates, and it assumes that he makes a rational decision to maximize his representation. Also for this voting rule, representativeness is computed by comparing the decisions taken by the committee, obtained with a weighted majority voting process, with the results of the direct popular vote. As can be appreciated in figure 7, our model is by far more efficient, reducing the size of the committees in more than a half.

Finally, we compare our method to an idealized perfect voting rule (PVR). This rule represents a situation of rational individuals that have a perfect knowledge of all the other individuals, which means that they perfectly know the opinion of all the other individuals. Moreover, they are globally networked, which means that they have a direct access to all other individuals, allowing their acts checked. In this situation, a voter indicates an individual which presents the higher overlap with his opinion vector. The selected committee is composed of first *F*_rep_⋅*N*_e_ individuals which poll more. Also in this case, the committee decisions are taken by means of a weighted majority vote. This voting rule, although unrealistic, is still useful, at least, in two respects. First, very small communities can exhibit similar characteristics. Second, the model is a useful yardstick for evaluating the levels of representativeness of other more realistic models.

Note that these voting rules can be considered, from a more abstract point of view, as an optimization problem: to find the vectors with greater overlap in an ensemble of given vectors, with no other constraints. By contrast, our original voting rule corresponds to the same optimization problem constrained by the fact that the overlap is inspected only locally, on a small subset of the ensemble of given vectors, because of the presence of the confidence circles.

In figure 7, we compare the representativeness of the PVR with our networked rule for different committee sizes. It is quite impressive that the representativeness of our voting rule is comparable with the PVR. Actually, when the number of opinions is relative small, the voting rule here presented performs even slightly better than the perfect rule. This effect disappears when the number of opinions is increased, and in this situation our networked rule is practically indistinguishable from the PVR.

## 4. Discussion

We introduced a general framework for studying the problem of selecting a committee of representatives to make decisions on behalf of a larger community. In our approach, we do not probabilistically study some properties of a particular voting rule, but we realistically implement it taking into account the effects of the individuals’ opinions and of their social relations. Moreover, we model the behaviour of the selected committee which is called to make choices about different issues. In this way, we are able, not simply to compare their choices with some abstract truth, but to clearly quantify the relation between representativeness and committee size.

Based on this scheme, it is possible to study the properties of a new networked voting rule, introduced with the aim of obtaining high representativeness together with strong accountability of the elected. This rule also presents the interesting feature that the candidates are not fixed in advance, but they emerge as a self-organized process.

The results of our simulations suggest that this rule is able to obtain a high representativeness for relatively small committees. In fact, these outputs are comparable with an ideal PVR, and they perform clearly better than a classical voting rule based on a closed list of candidates. Moreover, for fixed representativeness, the normalized committee size approximately scales with the inverse of the community size, allowing the scalability of this approach to very large populations. Finally, we were able to characterize the relation between the committee size and the representatives by means of a general inverse square root law.

These findings are robust and they are not strongly influenced by general properties of the network used to describe the individuals’ interactions. It seems that only the heterogeneity can have some role in modifying the relation between representativeness and committee size. In fact, the presence of a few individuals with very high connectivity can negatively influence the selection process of the committee. This fact is consistent with the risks related to a dominant position of single individuals, which may lead to a general sub-representation of the general opinion of the community.

The introduction of this networked voting rule, based on a decentralized large scale platform, can be considered as an interesting tool for implementing an hyper-representative mechanism of committee selection based on a distributed social mechanism where the use of a block chain encryption mechanism could guarantee the security of the voting process. This algorithm-based approach facilitates the participation of the entire population both as electors and as representatives, dismissing the importance of traditional authorities. Our analysis highlights the feasibility and potential of this election rule from the point of view of an organizational theory; specific and thorny political theory considerations and risks are not addressed.

## Data accessibility

The code used for this research is available at https://www.researchgate.net/publication/321804236_AxelDemX3.
